# Velocity Rescaling in Surface Hopping Based on Atomic Contributions to Electronic Transitions

**DOI:** 10.1021/acs.jctc.5c00737

**Published:** 2025-08-22

**Authors:** Eduarda Sangiogo-Gil, Lea M. Ibele, Richard Bleyer, Leticia González

**Affiliations:** † 27258 University of Vienna, Institute of Theoretical Chemistry, Währinger Str. 17, Vienna A-1090, Austria; ‡ 425798 Aix Marseille University, CNRS, ICR, Marseille 13397, France; § Research Platform on Accelerating Photoreaction Discovery (ViRAPID), University of Vienna, Vienna 1090, Austria

## Abstract

Surface hopping is a widely used method for simulating nonadiabatic dynamics, in which nuclear motion follows classical trajectories and electronic transitions occur stochastically. To ensure energy conservation during these transitions, atomic velocities must be adjusted. Traditional velocity rescaling methods either apply a uniform adjustment to atomic velocities, which can lead to size-consistency issues, or rely on nonadiabatic coupling vectors, which are computationally expensive and may not always be available. Here, we introduce two novel velocity rescaling methods that incorporate atomic contributions to electronic transitions, derived from the one-electron transition density matrix or the density difference between states for a given transition. The first method, *excitation-weighted velocity rescaling*, redistributes kinetic energy among atoms proportionally to their contributions to the electronic transition. This is achieved through a weighted scaling factor, computed from the population analysis of the one-electron transition density matrix or the density difference of the two states involved in the transition. The second method, *excitation-thresholded velocity rescaling*, adjusts the velocities only of atoms whose contributions exceed a predefined threshold, preventing unnecessary energy redistribution to atoms with minimal involvement in the excitation. We validate these approaches through excited-state dynamics simulations of fulvene and 1*H*-1,2,3-triazole. Our results show that excitation-weighted velocity rescaling closely reproduces the adjustments based on nonadiabatic coupling vectors for both fulvene and 1*H*-1,2,3-triazole.

## Graphical abstract

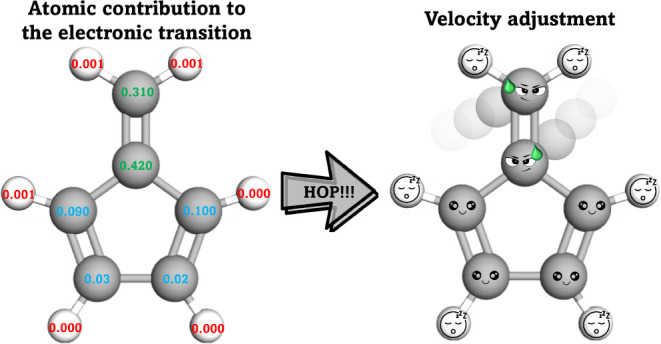

## 1 Introduction

The surface hopping (SH) method is a widely used approach for simulating nonadiabatic dynamics, where electrons are treated quantum mechanically and nuclei follow classical trajectories, bridging quantum and classical descriptions of molecular motion.^1–8^ The most commonly used version of SH—based on the fewest-switches algorithm—was introduced by Tully in 1990.^9^ This algorithm operates on the premise that nuclear motion is predominantly adiabatic, with nonadiabatic transitions occurring only momentarily and within localized regions of the configuration space. Tully proposed modeling these transitions as instantaneous “hops” between adiabatic potential energy surfaces (PESs). Within this framework, multiple independent trajectories are simulated, forming a statistical ensemble. The relative fraction of trajectories on each PES is used to approximate the population of each quantum state in a realistic dynamical process. The hops are not arbitrary: their probability is dictated by the electronic coupling between states and becomes significant in regions where the energy difference between PESs is small.^10,11^

The widespread success of SH over the past three decades stems from its simplicity and intuitive nature. The concept of nuclei evolving classically while stochastically transitioning between states makes the results easy to interpret. Additionally, SH is straightforward to implement computationally and, despite certain approximations, often yields reliable results across a variety of systems. One key advantage that makes it very attractive is its efficiency in handling large polyatomic systems, as calculations can be performed on-the-fly, eliminating the need for precomputed PESs and couplings.^12^ SH can also benefit from predefined PESs, such as linear vibronic coupling models (LVC), making the propagation in full dimensionality extremely efficient.^13–15^ The latter avoids the nontrivial task of selecting the most relevant modes in wavepacket propagation methods, while preserving high efficiency due to the predefined nature of the PESs. However, LVC models also impose limitations, as the linear coupling assumption around a reference geometry restricts their ability to capture anharmonic or large-amplitude nuclear motions. As a result, SH simulations based on LVCs are more suitable for studying photophysics (e.g., population dynamics) than photochemistry involving significant bond-breaking or structural rearrangements.

Nonetheless, the advantages of SH come with several approximations and adjustable parameters that can influence the resulting dynamics. Notable examples include the treatment of decoherence^16–20^ the algorithms for calculating hop probabilities,^9,21,22^ zero-point energy leakage correction,^23–26^ the choice of initial conditions,^26,27^ and the method for rescaling velocities after a hop.^28–33^ The latter is the focus of this study. When the system hops to a new state, the kinetic energy of the nuclei must be adjusted to ensure classical energy conservation. This adjustment is necessary because the new state has a different potential energy than the previous one, and the total energy (i.e., kinetic plus potential energy) needs to be constant. Accordingly, the kinetic energy is rescaled by modifying the velocity vector **v** such that the new kinetic plus potential energy equals the total energy of the previous time step, before the hop. This means that when a transition occurs between electronic states, in SH the velocities must be adjusted to conserve total energy. An approach commonly used is to rescale the velocity along its direction by a factor determined as

1 $$v^{\mathrm{new}}=v^{\mathrm{old}}\sqrt{\frac{E_{\mathrm{kin}}^{\mathrm{new}}}{E_{\mathrm{kin}}^{\mathrm{old}}}}$$

where $E_{\mathrm{kin}}^{\mathrm{new}}=E_{\mathrm{tot}}-E_{\mathrm{pot}}^{\mathrm{new}}$ , with *E*_tot_ being the total energy, $E_{\mathrm{pot}}^{\mathrm{new}}$ the potential energy of the new electronic state, and $E_{\mathrm{kin}}^{\mathrm{old}}$ is given by $E_{\mathrm{kin}}^{\mathrm{old}}=E_{\mathrm{tot}}-E_{\mathrm{pot}}^{\mathrm{old}}$ , where $E_{\mathrm{pot}}^{\mathrm{old}}$ is the potential energy before the hop.^2,6^ This approach is simple and computationally cheap, as it does not require additional quantities, such as nonadiabatic coupling vectors (NACVs), which are cumbersome to obtain. However, and critically, it introduces a *size-consistency* problem in the SH algorithm.^6,33^ The concept of size-consistency is reminiscent of electronic structure theory but can be used in an analogous way for nonadiabatic dynamics. Consider the following two scenarios: (*i*) a molecule *A*; and (*ii*) the same molecule *A*, but with a molecule *B* at an infinite distance. In the second case, since *A* and *B* do not interact, the dynamics of *A* should remain identical as in the first case. However, if a hop in *A* requires more energy than is available in its total energy, the results will differ. In case (*i*), where only *A* is present, the hop is “*frustrated*” and cannot occur. In case (*ii*), where *A* and *B* form a larger system, the total kinetic energy of the system may be sufficient to allow the hop. Here, rescaling the velocity of the entire system is unphysical, as the energy needed for the transition in *A* is effectively “borrowed” by slowing down atoms in *B*, despite *A* and *B* being noninteracting.

This size-consistency problem is not limited to infinitely separated systems. It also arises in finitely separated systems that contain weakly interacting subsystems,^34–36^ and it is particularly pronounced in large systems, such as chromophores in solution or in biological environments, where not all parts of the system are necessarily involved in the transition. For example, consider a system composed of multiple chromophores (such as those in refs 35 and 37–40 ). In such cases, the excitation can be localized in a subset of the chromophores, a single chromophore, or even a specific region of a chromophore. When SH dynamics are performed for these systems using the velocity rescaling presented in eq 1, the total kinetic energy of all the chromophores is available. This means that even if the excitation is strictly localized to a single chromophore or region, the system as a whole may have sufficient kinetic energy for a transition to occur. This could allow a hop to occur even when it is physically unmotivated, creating an artificial situation in which transitions occur that would not be plausible if the excitation were strictly confined. In simulations of systems with multiple chromophores, such as the 12-azobenzene chromophore setup in ref 35. the system’s total kinetic energy may always appear sufficient to allow a hop, even though the excitation is confined to one chromophore.

For simulations of chromophores in solution performed using a hybrid quantum mechanical/molecular mechanical (QM/MM) approach, it is common to consider only the kinetic energy of the atoms in the chromophore, which is typically the one molecule included in the QM region.^41^ However, the a large QM region can also encompass atoms that do not directly contribute to the electronic transition of interest.

A more systematic and size-consistent method, originally proposed by Tully, involves adjusting the velocity component along the NACV (see Appendix A).^28,42^ He demonstrated the physical motivation for this choice by deriving the Pechukas force—a semiclassical expression for the effective force that governs classical-like trajectory methods,^43^ such as Ehrenfest dynamics and SH, in a two-state quantum system that includes nonadiabatic effects. This force consists of three components: two aligned along the potential energy gradients of the current and target electronic states, and a third along the NACV. The latter, termed the transition force, is the only component proportional to the potential energy gap, ensuring energy conservation during the nonadiabatic transition.

While it is widely accepted that nuclear momentum (or, equivalently, nuclear velocity, since in surface hopping, momentum and velocity are related by momentum equal mass times the velocity. Since the mass is constant for each particle, rescaling the velocity is equivalent to rescaling the momentum) should be corrected along the NACV direction^9,29,33,44–47^ the direct computation of NACVs is often impractical. NACVs require the evaluation of wave function derivatives with respect to nuclear coordinates, which can be computationally expensive or even unavailable—as is the case in many electronic structure codes. It is worth noting that, although NACV are formally first-order derivatives of the electronic wave functions, their computation is not as widely available or as straightforward as that of energy gradients. NACVs involve cross-terms between different electronic states, ⟨ψ_*i*_|**∇**_**R**_ψ_*j*_⟩, and require careful phase tracking and overlap evaluations. While gradients are routinely available in many electronic structure methods, analytic NACVs are only implemented in a limited set of methods and are often more computationally demanding.

To circumvent this limitation, several SH algorithms avoid explicit NACV calculations when solving the electronic time-dependent Schrödinger equation.^48–50^ In this context, many SH implementations estimate the time-derivative couplings, $\left\langle \psi_{i}\right|\frac{\partial \psi_{j}}{\partial t}\rangle =\frac{\partial R}{\partial t}\left\langle \psi_{i}\right|\nabla_{R}\psi_{j}\rangle =v\cdot d_{ij}$ using numerical differentiation.^51,52^ A more robust alternative involves the evaluation of wave function overlaps between consecutive time steps. Among these strategies, one of the most widely employed is the local diabatization scheme,^53,54^ which circumvents the explicit calculation of nonadiabatic coupling vectors by constructing a diabatic-like basis from the overlap matrix of adiabatic states at two successive nuclear geometries. In such cases, the most common procedure for rescaling nuclear velocities after a surface hop follows eq 1.

Recently, Toldo et al.^33^ systematically investigated various velocity adjustment schemes in SH dynamics. They assessed the gradient difference method as an approximation for the NACV and explored an alternative approach that reduces the available kinetic energy by distributing it across the system’s vibrational degrees of freedom before a nonadiabatic transition. The latter approach rescales velocities along the momentum direction; however, it mitigates the occurrence of so-called “unavoidable” back-hoppings by reducing the kinetic energy available for hops. However, it introduces size-consistency issues, as the uniform distribution of kinetic energy among all vibrational modes can slow down the motion of the degrees of freedom that are not directly involved in the transition.

In order to overcome the size-consistency problem while ensuring a physically meaningful redistribution of velocity, here we propose a method that adjusts nuclear velocities based on atomic contributions to the electronic transition. These contributions are derived from either the one-electron transition density matrix or the electron density difference between the two involved electronic states.^55^ The one-electron transition density matrix and the electron density difference provide essential insights into the spatial characteristics of electronic transitions.^56^ Since these quantities are readily available in many electronic structure methods, our approach is widely applicable. By applying population analysis to those matrices, we can identify the most relevant atoms involved in the transition and redistribute kinetic energy accordingly. This ensures that velocity rescaling remains size-consistent and physically meaningful while minimizing unnecessary adjustments to unrelated degrees of freedom.

Accordingly, this paper introduces two velocity rescaling methods based on atomic contributions. The first method is weighted per-atom velocity rescaling, where the kinetic energy is rescaled per atom according to its excitation weight derived from the one-electron transition density matrix or the density difference matrix. A correction factor ensures energy conservation while maintaining physically meaningful momentum adjustments. The second method is a simpler approach based on threshold-based velocity rescaling. This scheme adjusts only the velocities of atoms whose contributions exceed a predefined excitation threshold. By applying velocity rescaling solely to those atoms actively participating in the electronic transition, the redistribution of kinetic energy remains localized to the most relevant nuclear degrees of freedom, preserving both physical accuracy and computational efficiency. The new methods are tested on the excited-state dynamics of the fulvene molecule (Figure 1, left), because previous studies have shown that population decay in the *S*_1_ state is strongly influenced by the choice of velocity rescaling method in SH.^30,31,33,44^ Fulvene is therefore a prototype system to validate the methodologies proposed. Additionally, we examine the excited-state dynamics of 1*H*-1,2,3-triazole (triazole) in order to validate our approach on a system containing heteroatoms (Figure 1, right).

**Figure 1:**
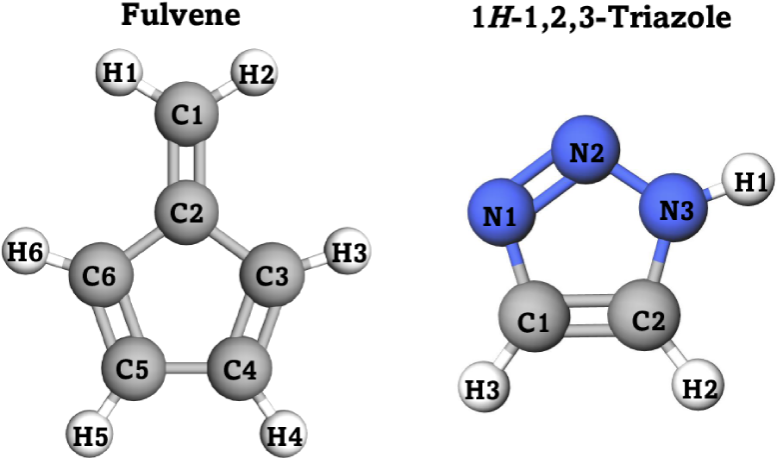
Structures of fulvene (left) and 1*H*-1,2,3-triazole (right). Carbon atoms are shown in gray, hydrogen in white, and nitrogen in blue.

The remainder of the paper is structured as follows. Next, we briefly introduce the population analysis used to identify the atomic contributions to electronic transitions and then explain the two proposed velocity rescaling methods. The excited-state dynamics for fulvene and triazole are presented, focusing on the impact of the velocity rescaling algorithm on electronic and nuclear dynamics. Finally, we summarize our findings.

## 2 Methodology

### 2.1 Atomic Contributions to Electronic Transitions

We assess how individual atoms contribute to an electronic transition by assigning atomic charges associated with the redistribution of electron density. To this end, we use two complementary approaches.

#### 2.1.1 Approach 1: Electron and Hole Decomposition

In the first approach, we decompose the transition into electron and hole components, which can be analyzed independently. This decomposition can be performed on either the one-electron transition density matrix (1TDM) or the density difference (DD) matrix. For clarity, we denote both generically as **T**. Both matrices are valid choices, and we have evaluated both in our analysis.

Within multiconfigurational frameworks—consistent with the methodology employed in this study—the electron and hole transition density matrices (**D** ^e^ and **D** ^h^) are defined as

2 $$D_{pq}^{e}=\underset{r}{\sum }T_{rp}T_{rq},,D_{pq}^{h}=\underset{r}{\sum }T_{pr}T_{qr}$$

where *p*, *q*, *r* index the active space orbitals.

To obtain atomic contributions, we perform a Löwdin population analysis on both **D** ^e^ and **D** ^h^. The Löwdin scheme is chosen for its orthogonalization properties, which improve interpretability and reduce (though not eliminate) basis set dependence.^57^ This analysis yields atomic electron and hole charges, **q** ^e^ and **q** ^h^.

The total contribution of an atom *A* to the transition is computed as

3 $$q_{A}=\left|q_{A}^{e}\right|+\left|q_{A}^{h}\right|$$

The use of absolute values ensures that the electron and hole contributions do not cancel, thus reflecting the full extent of atomic participation.

We refer to this method as *qeh*. If based on the 1TDM, it is labeled qeh*/*1*TDM*; if based on the DD matrix, it is labeled *qeh/DD*.

#### 2.1.2 Approach 2: Transition Charge Analysis

In the second approach, we apply a Löwdin analysis directly to the 1TDM to obtain transition charges, **q** ^tr^.^56,58^ Each atom’s contribution to the excitation is then:

4 $$q_{A}=\left|q_{A}^{\mathrm{tr}}\right|$$

This method is referred to as *qtr*.

Although we exclusively use Löwdin charges in this work, other definitions are also feasible. For example, one could compute the electrostatic potential from a given density matrix (e.g., the 1TDM) and fit atomic charges to reproduce it. Exploring the effects of these alternative schemes is left for future work.

### 2.2 Excitation-Weighted Velocity Rescaling (**v**_*w*_)

This method redistributes the available kinetic energy after a surface hop according to each atom’s contribution to the electronic transition.

We first define a normalized weight for each atom *A*:

5 $$c_{A}=\frac{q_{A}}{\sqrt{\underset{B}{\sum }q_{B}^{2}}}$$

where *q*_*A*_ is the atomic contribution from either eq 3 or eq 4.

The total kinetic energy available to the system undergoing an electronic transition is

6 $$E_{\mathrm{kin}}^{\mathrm{avail}}=\frac{1}{2}\underset{A}{\sum }w_{A}M_{A}v_{A}^{2}$$

where $w_{A}=c_{A}^{2}$ , *M*_*A*_ is the atomic mass of atom *A*, and **v**_*A*_ is the velocity of atom *A*.

To redistribute this energy, we define a per-atom velocity scaling factor:

7 $$f_{A}=\sqrt{\frac{E_{\mathrm{kin}}^{\mathrm{new}}}{E_{\mathrm{kin}}^{\mathrm{old}}}}\times \left[1+\alpha \left(w_{A}-\mathrm{mean}\left(w\right)\right)\right]$$

Here, α is a tuning parameter controlling the strength of the weighting. If α = 0, the scaling is uniform and corresponds to eq 1. Larger α values emphasize atoms with higher contributions.

However, directly scaling velocities by *f*_*A*_ alone does not conserve total kinetic energy. To ensure energy conservation, we first compute the kinetic energy associated with the scaled velocities:

8 $$E_{\mathrm{kin}}^{*}=\frac{1}{2}\underset{A}{\sum }M_{A}{\left(f_{A}v_{A}\right)}^{2}$$

We then apply a global normalization factor:

9 $$N=\sqrt{\frac{E_{\mathrm{kin}}^{\mathrm{new}}}{E_{\mathrm{kin}}^{*}}}$$

(note that the kinetic energy after the hop is calculated as $E_{\mathrm{kin}}^{\mathrm{new}}=E_{\mathrm{tot}}-E_{\mathrm{pot}}^{\mathrm{new}}$ ). The final rescaled velocities are given by

10 $$v_{A}^{\mathrm{new}}=v_{A}^{\mathrm{old}}\times f_{A}\times N$$

### 2.3 Excitation-Thresholded Velocity Rescaling (**v**_*t*_)

This method restricts velocity rescaling to atoms with significant contributions to the excitation. An atom is considered significant if its contribution satisfies:

11 $$q_{A}>n\times \max\left(q\right)$$

where *n* ∈ [0, 1] is a user-defined threshold and *q*_*A*_ is the atomic contribution from either eq 3 or eq 4.

After a surface hop, velocity rescaling is applied only to the subset of atoms that meet this criterion, using the standard rescaling procedure (see eq 1). This targeted redistribution ensures that kinetic energy adjustments are limited to the atoms most involved in the electronic transition.

### 2.4 Summary of the Methods

In total, we have defined three approaches to determine atomic contributions to a given electronic transition: one based on electron–hole charges derived from the 1TDM (*qeh/*1*TDM*), another using electron–hole charges from the DD matrix (*qeh/DD*), and a third based on transition charges (*qtr*).

Additionally, we have proposed two approaches for velocity rescaling after a hop: the excitation-weighted rescaling (**v**_*w*_) and the excitation-thresholded rescaling (**v**_*t*_). Each of these rescaling methods can be combined with any of the three charge analysis approaches, resulting in six possible method variants, see Table 1. For example, applying excitation-thresholded rescaling with the electron–hole charges computed from the 1TDM is denoted as **v**_*w*_(*qeh/*1*TDM*).

**Table 1:** Summary of the Methods Resulting from the Combination of Two Different Velocity Rescaling Schemes—**v**_*w*_ and **v**_*t*_—with Three Approaches to the Determination of Atomic Contributions to Electronic Transitions: q*eh/*1*TDM*, *qeh/DD*, and *qtr*

	*qeh/*1*TDM*	*qeh/DD*	*qtr*
**v**_*w*_	**v**_*w*_(*qeh/*1*TDM*)	**v**_*w*_(*qeh/DD*)	**v**_*w*_ *(qtr)*
**v**_*t*_	**v**_*t*_(*qeh,* 1*TDM*)	**v**_*t*_(*qeh*, *DD*)	**v**_*t*_(*qtr*)

## 3 Validation Systems

Our approach is validated on two representative systems: fulvene and 1*H*-1,2,3-triazole (Figure 1). The choice of fulvene is motivated by the previous work of Ibele and Curchod^44^ who reported significant differences in the nonadiabatic dynamics when using SH, depending on whether the velocity adjustment was made along the velocity direction (eq 1) or the NACV direction. These observations were further supported by Toldo et al.^33^ who explored additional algorithms for velocity rescaling. Fulvene’s small size and ultrafast excited-state relaxation dynamics make it an ideal benchmark for testing novel approaches to nonadiabatic dynamics.^33,44,59^ The second system is 1*H*-1,2,3-triazole, a five-membered aromatic heterocycle containing three nitrogen atoms. This molecule is known for its rich photochemistry and ultrafast nonradiative decay pathways.^60–62^ Previous studies have shown that its *S*_1_ excited state is dissociative, leading to elongation and rupture of the N2–N3 bond. This process relaxes the system to the ground state, potentially releasing molecular nitrogen (*N*_2_) and forming products such as ethanimine.^60,61^

We emphasize that the primary objective of our study is not to provide a comprehensive mapping of the photochemical behavior or product distribution of 1*H*-1,2,3-triazole, but rather to evaluate the performance of our velocity rescaling algorithm within the first 150 fs of excited-state dynamics.

Although the two approaches proposed for velocity rescaling (**v**_*w*_ and **v**_*t*_) can be particularly suited for larger systems, here we prioritized assessing its performance and generalizability in small molecules like fulvene and triazole.

Specifically for fulvene, we tested three variants of the excitation-weighted rescaling method (Section 2.2): **v**_*w*_(*qeh/*1*TDM*), using α = 0.25, 0.50, and 1.00 (see eq 7 for the definition of the α parameter); **v**_*w*_(*qeh/DD*), using α = 0.50 and 1.00; and **v**_*w*_ *(qtr)*, using α = 0.50 and 1.00. We also considered one variation of the excitation-thresholded method, **v**_*t*_(*qeh*, 1*TDM*), which applies a threshold of *n* = 0.30 (see eq 11). In addition, we benchmarked our approaches (**v**_*w*_ and **v**_*t*_) against two commonly used methods: velocity adjustment along the NACV direction (**d**) and the standard velocity rescaling method described in eq 1 (**v**_full_). We further compared our simulations with results from the ab initio multiple spawning (AIMS) method,^63–65^ which provides a robust reference due to its insensitivity to velocity rescaling schemes.

For 1*H*-1,2,3-triazole, we applied the same three variants of the excitation-weighted rescaling method — **v**_*w*_(*qeh/*1*TDM*), **v**_*w*_(*qeh/DD*), and **v**_*w*_ *(qtr)* — using α = 0.50 and α = 1.00 for each approach, and compared the results with both the NACV-based velocity adjustment (**d**) and the standard rescaling method (**v**_full_).

## 4 Computational Details

### 4.1 Electronic Structure

The electronic properties required to propagate the nuclear dynamics of fulvene—including energies, nuclear gradients, and NACVs—were computed at the SA(3)-CASSCF­(6,6)/6-31G* level of theory. The active space comprised six electrons distributed over six molecular orbitals, encompassing the full π system: three pairs of π and π* orbitals (see Figure S1).

For triazole, the calculations were performed at the SA(7)-CASSCF­(10,8)/6-31G* level, with an active space of ten electrons in eight orbitals: two nonbonding (*n*) orbitals, two pairs of π and π* orbitals, and one pair of σ and σ* orbitals localized on the N2–N3 bond (see Figure S2). Initially, the SA(5)-CASSCF­(10,8)/6-31G* level was tested for triazole. However, many trajectories failed early in the simulations due to convergence issues. To improve stability, we increased the number of averaged states to SA(7), which resolved these problems and allowed the simulations to proceed reliably.

All electronic structure calculations were performed using the OpenMolcas program, version 23.10 for SH dynamics and version 24.10 for AIMS dynamics.^66^

Population analyses were carried out using the internal WFA module in OpenMolcas. This module directly yields the transition charges (eq 4), as well as the hole and electron charges (eq 3), computed from both the 1TDM and the DD matrix.

### 4.2 Nonadiabatic Dynamics

Initial coordinates and velocities were stochastically sampled from a Wigner distribution corresponding to uncoupled harmonic oscillators, constructed from a harmonic frequency analysis at the ground state optimized geometry. For fulvene, 200 initial conditions—comprising geometries, velocities, and initial electronic states—were selected within an excitation energy window of 3.7–4.3 eV, targeting excitation to the *S*_1_ state (see absorption spectrum in Figure S5). The nuclear dynamics were propagated with a time step of 0.2 fs for a total simulation time of 50 fs. For triazole, 119 initial conditions were selected based on an excitation energy range of 5.75–6.25 eV, also corresponding to excitation to the *S*_1_ state (see Figure S6). The nuclear dynamics were propagated with a time step of 0.2 fs for a total simulation time of 150 fs.

For each system, all SH simulations were launched from the same set of geometries and velocities. To ensure stochastic consistency across simulations, different velocity rescaling schemes were applied using identical random seeds. The fewest switches algorithm was used in the SH simulations, incorporating the local diabatization approach^53,54^ for propagating the electronic coefficients (even when NACVs were employed for velocity adjustment) with the Granucci-Persico energy-based decoherence correction^17^ using the empirical parameter of 0.1 au. In cases of frustrated hops, the momentum direction was preserved. The SH simulations were propagated using the SHARC molecular dynamics package.^67^

The AIMS dynamics on fulvene were performed for a subset of 170 initial conditions (geometries, velocities, and initial electronic states) drawn from the SH ensemble. The quantum amplitudes were propagated with a full diagonal propagator in the adiabatic basis with norm-preserving interpolation time-derivative couplings.^68^ A time step of 10 atomic time units (approximately 0.24 fs) was used and the absolute value of the time-derivative coupling above 0.0039 au–1 was used as a spawning criterion. The velocity rescaling of newly spawned trajectories was performed for the full velocity vector, as it was observed in previous studies^44^ that this has no effect on AIMS dynamics. The width of the Gaussians was chosen to be 4.7 and 22.7 1/bohr^2^ for H and C, respectively. The AIMS simulations were run with the PySpawn code.^69–71^

## 5 Results and Discussion

### 5.1 Fulvene

The photodynamics of fulvene have been extensively studied in the literature.^33,44,48,59,72–76^ Upon photoexcitation to the *S*_1_ state, it undergoes ultrafast excited-state decay through two primary nonadiabatic pathways mediated by conical intersections with the *S*_0_ state. The dominant pathway is driven by the stretching of the C1–C2 bond (see Figure 1), leading to a strongly sloped conical intersection. The nuclear wavepacket passes through this intersection, undergoes reflection, and recrosses, resulting in a stepwise decay of the *S*_1_ population. The alternative pathway involves a twist of the same C1–C2 bond, where a fully twisted geometry corresponds to a peaked conical intersection, while a partial twist results in a smoother, less pronounced slope. An extended *S*_1_/*S*_0_ seam enables population transfer across a range of geometries, from planar to highly twisted. Simulations and theoretical studies consistently indicate that most of the population decays via the sloped conical intersection, while a smaller fraction follows the twisting mechanism. Therefore, in terms of nuclear dynamics, the most important internal coordinate to investigate is the C1–C2 bond length, followed by the two cis dihedrals (C3–C2–C1–H2 and C6–C2–C2–H1) and the two trans dihedrals (C3–C2–C1–H1 and C6–C2–C2–H2). In Figure 2, we present the *S*_1_ state population decay calculated using SH with different velocity adjustments after a hopping event, as well as for AIMS. Specifically, we compare velocit*y*-direction adjustment using the full kinetic energy available (**v**_full_), NACV-direction adjustment (**d**), the three different variants of excitation-weighted velocity rescaling (**v**_*w*_), and excitation-thresholded velocity rescaling (**v**_*t*_(*qeh,* 1*TDM*)).

**Figure 2:**
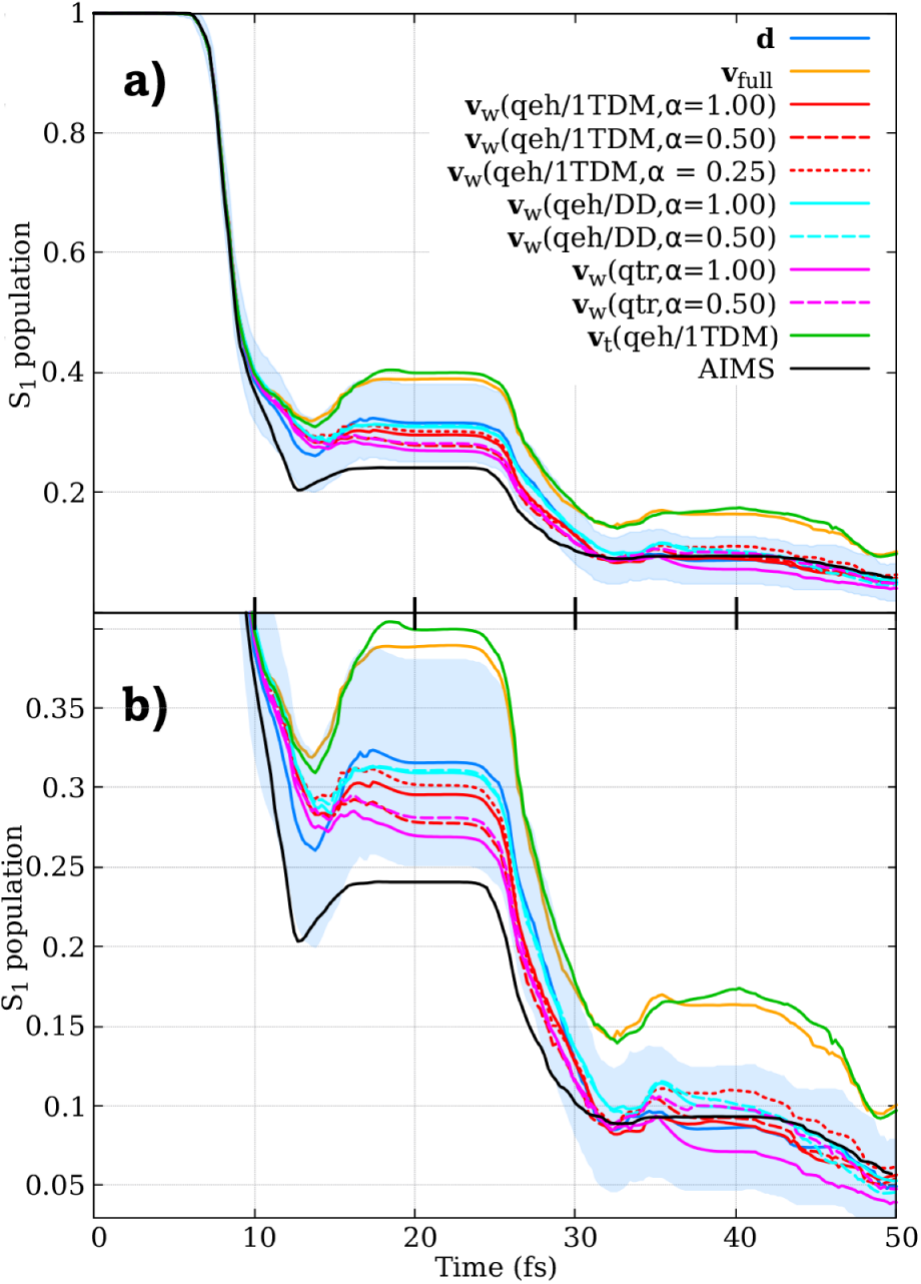
(a) Time-resolved *S*_1_ state population of fulvene over the first 50 fs, computed using surface hopping with different velocity adjustment algorithms: velocit*y*-direction adjustment using the full kinetic energy available (**v**_full_), NACV-direction adjustment (**d**), three different flavors of the excitation-weighted velocity rescaling (**v**_*w*_), and excitation-thresholded velocity rescaling (**v**_*t*_(*qeh,* 1*TDM*)). The *S*_1_ population, obtained with ab initio multiple spawning (AIMS) is also showed. Shaded regions indicate 95% confidence intervals (Γ) for the NACV-direction adjustment results, calculated as $\Gamma =p\pm 1.96\times \sqrt{\frac{p\left(1-p\right)}{N_{\mathrm{traj}}}}$ , where *p* is the state population and *N*_traj_ = 197 is the number of trajectories. (b): Magnified view of the *S*_1_ population across all approaches.

Upon excitation to the *S*_1_, the energy of the *S*_1_ and *S*_0_ states approaches each other, reaching a conical intersection after 7 fs, which effectively transfers about 80% of the population from *S*_1_ to *S*_0_. However, after approximately 12 fs, a back transfer of population to the *S*_1_ state is observed. The coupling region is encountered again after 24 fs. At around 24 fs, the population decays again to the *S*_0_ state, and after approximately 32 fs, a second repopulation of the *S*_1_ state is observed, though less pronounced this time.

All the simulations exhibit a similar initial population decay. The recrossing and back transfer to *S*_1_ occur in all approaches, but the extent of population transfer depends strongly on the nonadiabatic dynamics method employed. While AIMS predicts that only about 24% of the population remains in *S*_1_ between 16 and 24 fs, the repopulation process in SH occurs slightly later, leading to higher excited-state population plateaus. The final *S*_1_ population reaches approximately 31% for the **d** adjustment, 29% for the **v**_*w*_(*qeh/*1*TDM*) adjustment, 31% for the **v**_*w*_(*qeh/DD*) adjustment, 27% for the **v**_*w*_ *(qtr)* adjustment, 40% for the **v**_*t*_(*qeh,* 1*TDM*) adjustment, and 39% for the **v**_full_ adjustment.

By using the **d** adjustment as a reference for SH dynamics, we observe that both **v**_full_ and **v**_*t*_(*qeh,* 1*TDM*) significantly overestimate the repopulation to the *S*_1_ state, as previously noted in refs 33 and 44. The **v**_*w*_ approaches underestimate the repopulation in comparison to **d** adjustment; however, **v**_*w*_(*qeh/*1*TDM*) and **v**_*w*_(*qeh/DD*) show much better agreement with the **d** dynamics, representing a notable improvement in the population dynamics. It is gratifying to see that for all **v**_*w*_ approaches, the *S*_1_ population over the entire time interval of the dynamics falls within the 95% confidence interval (shaded region in Figure 2) of the **d** dynamics, indicating consistency between these methods within statistical uncertainty.

In addition, we have analyzed the internal consistency between the classical populations (Figure S7) and the quantum populations (Figure 2), and from this comparison, one can observe that internal consistency is preserved. Note that the classical populations are computed as *N*_*i*_(*t*)/*N*_*traj*_, where *N*_*i*_(*t*) is the number of trajectories in which the active state at time *t* is *i*, and *N*_*traj*_ is the total number of trajectories. In contrast, the quantum populations are calculated as the average over all trajectories of the state probabilities |*C*_*i*_(*t*)|^2^, where *C*_*i*_(*t*) are the electronic adiabatic coefficients.

We use AIMS dynamics as a benchmark, as it has been previously shown that it is insensitive to rescaling schemes.^44,75^ One of the reasons for this observation is the standard spawning algorithm^65,77^ as implemented in PySpawn, as it ensures that the new trajectory is created at the point of maximum coupling, which generally is expected to coincide with the point of minimal energy difference which also minimizes the extend of kinetic energy rescaling. The difference between the SH and AIMS population dynamics is particularly evident during the first decay, where the AIMS S_1_ population drops to 20% after 12 fs. The subsequent repopulation up to 25% in AIMS is reproduced in the SH simulations that use **d** or **v**_*w*_ approaches. The AIMS population trace at this point does not lie within the 95% confidence interval of the **d** dynamics; however, this discrepancy is primarily due to differences in the initial deactivation behavior (within the first 12 fs). It is important to note that the first deactivation is minimally, if at all, affected by the choice of rescaling scheme after a hop or spawn as the rescaling only affects the behavior of the trajectory after the hop and does not alter the initial hopping probability to energetically lower lying states. Therefore, the observed deviations can be attributed mainly to differences in the number of initial conditions, the degree of convergence achieved, and the fundamental conceptual distinctions between the SH and AIMS methods. Despite these differences, the excellent agreement in the repopulation phase between the **v**_*w*_ dynamics and AIMS serves as strong validation for the velocity rescaling scheme.

Table 2 presents the total number of hops (*S*_1_ → *S*_0_ transitions), back-hops (*S*_0_ → *S*_1_ transitions), and frustrated hops across all SH simulations. Additionally, we report the total number of trajectories considered in our analysis. Trajectories where total energy conservation was violated (total energy variation was superior to |0.20| eV) or where issues in the electronic structure calculations occurred were excluded. To provide a normalized comparison, values in parentheses represent the number of hops, back-hops, and frustrated hops events per trajectory, e.g., $\frac{\mathrm{Number of hops}}{\mathrm{Number of trajectories}}$ .

**Table 2:** Number of Hops (*N*_hop_), Back-Hops (*N*_back hop_), Frustrated Hops (*N*_frust_), and Total Trajectories Considered (*N*_traj_) for Fulvene under Different Velocity Adjustment Schemes Following a Hopping Event^a^ ^b^

	*N*_hop_	*N*_back hop_	*N*_frust_	*N*_traj_
**d**	226 (1.147)	39 (0.198)	52 (0.264)	197
**v**_full_	260 (1.300)	82 (0.410)	0 (0.000)	200
**v**_*w*_(*qeh/*1*TDM*, α = 1.00)	225 (1.148)	41 (0.209)	53 (0.270)	196
**v**_*w*_(*qeh/*1*TDM*, α = 0.50)	226 (1.141)	39 (0.197)	53 (0.268)	198
**v**_*w*_(*qeh/*1*TDM*, α = 0.25)	228 (1.146)	42 (0.211)	61 (0.306)	199
**v**_*w*_(*qtr*, α = 1.00)	226 (1.147)	37 (0.188)	48 (0.244)	197
**v**_*w*_(*qtr*, α = 1.00)	230 (1.156)	41 (0.206)	43 (0.216)	199
**v**_*w*_(*qeh/DD*, α = 1.00)	235 (1.187)	47 (0.237)	48 (0.242)	198
**v**_*w*_(*qeh/DD*, α = 0.50)	232 (1.184)	45 (0.229)	47 (0.240)	196
**v**_*t*_(*qeh,* 1*TDM*)	257 (1.318)	83 (0.426)	1 (0.005)	195

a The velocity adjustments include velocity-direction adjustment using the full kinetic energy reservoir (**v**_full_), adjustment along the nonadiabatic coupling vectors (**d**), various flavors of excitation-weighted velocity rescaling (**v**_w_), and excitation-thresholded velocity rescaling (**v**_t_(*qeh*, 1*TDM*)).

b Values in parentheses indicate the number of hopping, back hopping , and frustrated hopping events per trajectory (e.g., $\frac{N_{\mathrm{hop}}}{N_{\mathrm{traj}}}$ ).

The results clearly indicate that the increased repopulation of the *S*_1_ state observed with **v**_full_ and **v**_*t*_(*qeh,* 1*TDM*) is primarily due to an excessive number of back-hopping events. Specifically, **v**_full_ and **v**_*t*_(*qeh,* 1*TDM*) exhibit 0.410 and 0.426 back-hops per trajectory, respectively—substantially higher than those observed for the other methods, which do not exceed 0.229. This suggests that these approaches provide excess kinetic energy for nonadiabatic transitions.

In contrast, the **d** adjustment, as well as the excitation-weighted velocity rescaling (**v**_*w*_) methods, significantly reduce the number of back-hops. The reduction in back-hopping for these approaches can be attributed to the lower available kinetic energy for hopping, as constrained by their respective rescaling mechanisms. The derivation of the kinetic energy available when using NACV to adjust velocities is provided in Appendix A (see eq 22). Overall, our results suggest that the **d** and **v**_*w*_ approaches provide a more balanced description of nonadiabatic transitions by reducing spurious back-hoppings. A key observation is that the excitation-weighted velocity rescaling (**v**_*w*_) induces a hopping behavior that closely resembles the **d** approach. The **v**_*w*_ methods not only maintain a lower number of back-hops but also introduce a controlled number of frustrated hops, preventing excessive transitions and thus better agreement with physically realistic nonadiabatic dynamics.

To compare the kinetic energy available for hopping, we extracted the back-hopping geometries and velocities from simulations using the **d** adjustment. We then computed the available kinetic energy using the full kinetic energy reservoir (**v**_full_), adjustment along the nonadiabatic coupling vectors (**d**), various flavors of excitation-weighted velocity rescaling (**v**_*w*_), and excitation-thresholded velocity rescaling (**v**_*t*_(*qeh,* 1*TDM*)). The results are presented in Figure 3, where each *x*-component corresponds to a different geometry. We analyzed a total of 38 geometries.

**Figure 3:**
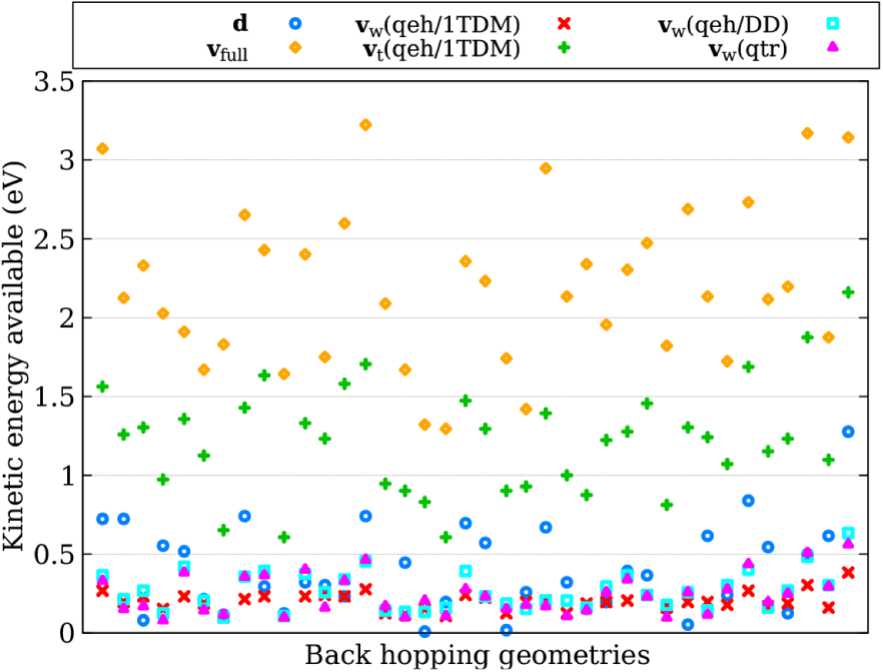
Kinetic energy available for hopping across different approaches for fulvene. Each *x*-component corresponds to a back-hopping geometry and velocity extracted from simulations using the NACV-direction adjustment. The kinetic energy is computed using four different methods: **d** (eq 22), **v**_*w*_ (eq 6), the full kinetic energy reservoir **v**_full_, and **v**_*t*_, which considers only the atoms contributing most to the electronic transition when computing the kinetic energy.

As we can see, the kinetic energy available for hopping in the **v**_*w*_ approach is often underestimated compared to that obtained using the NACV-direction adjustment (**d**). However, it shows better agreement than the other approaches. For both **d** and **v**_*w*_, the kinetic energy available for hopping remains below 1.0 eV (except for one geometry in the **d** method). In contrast, when considering the full kinetic energy reservoir (**v**_full_), the available kinetic energy is typically above 1.5 eV. When focusing only on atoms that contribute the most to the electronic transition to compute the kinetic energy (**v**_*t*_(*qeh,* 1*TDM*)), we observe a significant improvement; however, in many cases, the values are still underestimated compared to the **d** adjustment.

Although the results obtained for the electronic dynamics are quite satisfactory, the first proposed method (**v**_*w*_) prompted us to investigate whether it also captures and improves the nuclear dynamics compared to, for instance, **v**_full_. To this end, in Figure 4 we plot the time evolution of the average bond length between atoms C2 and C1 of fulvene (recall Figure 1), obtained from SH with different velocity adjustment algorithms. Additionally, Figure 4b shows the difference in bond length relative to the **d** simulations. We observe that the C1–C2 stretching oscillates more slowly when using the **v**_full_ method compared to the others. This behavior is expected, as **v**_full_ redistributes the total energy equally across all atoms, without considering their individual contributions to the nonadiabatic process. In contrast, the NACV-direction adjustment and excitation-weighted velocity rescaling methods (**v**_*w*_) account for the varying atomic contributions, leading to a more localized energy redistribution. Since the C1–C2 stretching mode is known to actively participate in the *S*_1_/*S*_0_ transition, a stronger local adjustment enhances the kinetic energy in this specific coordinate, resulting in faster oscillations of the C1–C2 bond.

**Figure 4:**
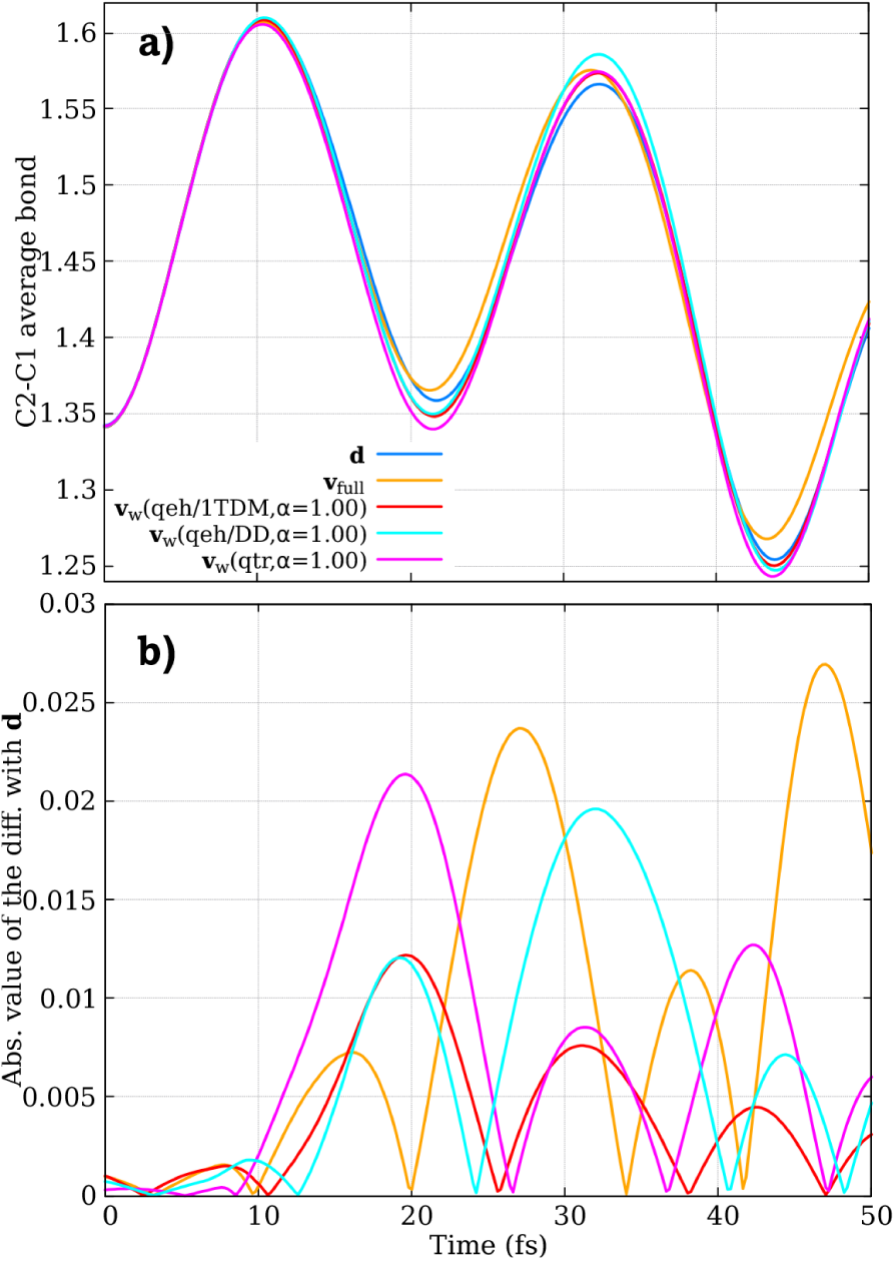
(a) Time evolution of the average bond length between atoms C2 and C1 of fulvene (see Figure 1), computed using surface hopping with different velocity adjustment algorithms: velocit*y*-direction adjustment using the full kinetic energy reservoir (**v**_full_), NACV-direction adjustment (**d**), and excitation-weighted velocity rescaling (**v**_*w*_). (b) Difference in bond length relative to the **d** simulations.

Importantly, the **v**_*w*_ also appears to enhance nuclear dynamics, as it preferentially distributes energy to atoms actively involved in electronic transitions while minimizing unnecessary energy redistribution to those that are not. This seems to improve the size-consistency problem associated with the SH approach using **v**_full_, without requiring explicit NACV calculations.

Next, we analyzed the normalized contribution of the NACV between the *S*_1_/*S*_0_ states per atom for various back-hopping geometries extracted from **d** adjustment simulations, using the same geometries as in Figure 3. We then computed the average of these coefficients across all geometries and compared them to the average coefficients obtained from eq 5 for the different **v**_*w*_ approaches.

Figure 5 compares the average normalized contribution of the NACV per atom for the different back-hopping geometries extracted from simulations using the **d** adjustment with the average coefficients calculated according to eq 5 for the various **v**_*w*_ approaches. The *x*-axis represents the atoms (see Figure 1). The *y*-axis shows the average of the coefficients (eq 5) and the normalized nonadiabatic coupling contribution from the **d** dynamics for the 38 back-hopping geometries obtained through **d** calculations. Our objective is to assess whether the atomic contributions used to adjust the velocity in **v**_*w*_ align with those in the **d**. From Figure 5, we observe that the individual atomic contribution **v**_*w*_(*qeh/DD*) aligns best with the atomic contribution obtained from **d**, and in general, the qualitative agreement is satisfactory.

**Figure 5:**
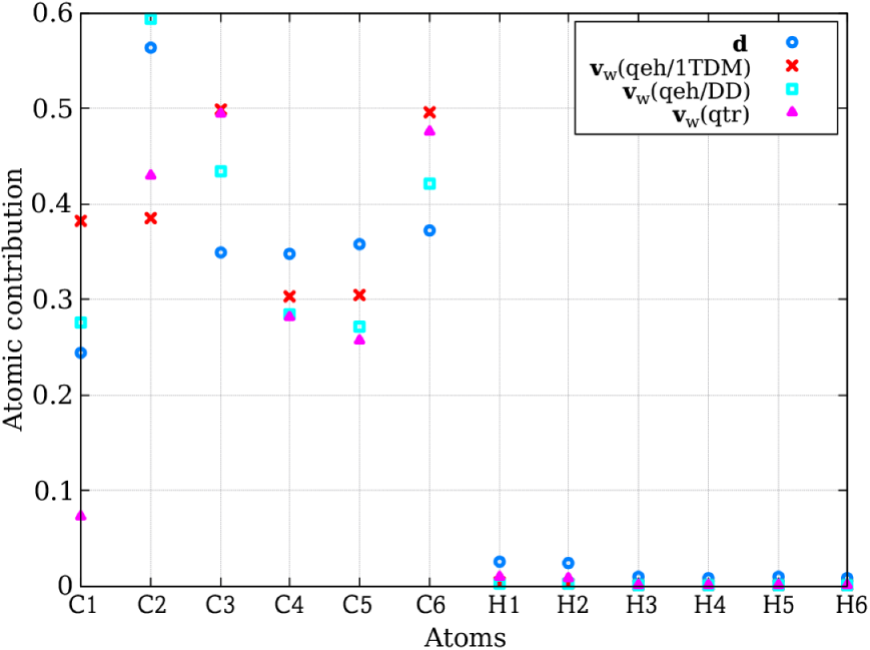
Comparison of the atomic contribution in the velocity adjustment for the **d** and **v**_*w*_ in fulvene’s dynamics. We present the average normalized contribution of the NACV per atom for different back-hopping geometries extracted from simulations using the **d** adjustment is compared with the average coefficients calculated according to eq 5 for the different **v**_*w*_ approaches. The *x*-axis represents individual atoms (see Figure 1). The *y*-axis shows the average coefficient values and the normalized nonadiabatic coupling contribution for the 39 back-hopping geometries obtained through **d** calculations.

As mentioned previously, the dominant pathway is driven by the stretching of the C1–C2 bond. For **v**_*w*_(*qeh/DD*), the contributions from C1 and C2 are slightly stronger than those from **d**. This may explain why the stretching bond oscillations shown in Figure 4 are somewhat stronger in **v**_*w*_(*qeh/DD*) compared to **d**. For **v**_*w*_(*qeh/*1*TDM*), the contribution from C1 is overstated, while the contribution from C2, which has the highest contribution in **d**, is understated. This discrepancy may lead to a cancellation error, but the oscillation of the C1–C2 stretching shows good agreement with **d**.

Using **v**_*w*_(*qtr*), the contributions from C1 and C2 are both understated. As shown in Figure 4, the oscillations of this bond exhibit a slight delay, particularly at longer times. This effect is even more pronounced with the **v**_full_ approach, where the oscillations are substantially delayed compared to other methods due to the equal distribution of kinetic energy among all atoms.

In summary, regarding electronic dynamics, the **v**_*w*_ method, in particular the **v**_*w*_(*qeh/DD*), demonstrates substantial improvement compared to the other approaches, aligning closely with the NACV-direction adjustment. Additionally, we observe that the electronic dynamics remain robust with respect to the chosen α value. However, the electronic dynamics obtained with the **v**_*t*_ method is quite similar to that observed with **v**_full_, especially because the system still retains excess kinetic energy, resulting in an excessive number of back-hoppings. We note that the threshold-based velocity rescaling procedure is inherently system-dependent. In small molecules such as fulvene, where the kinetic energy contributions from heavy atoms (e.g., carbon) are relatively uniform and the influence of light atoms (e.g., hydrogen) is non-negligible, the method offers limited improvement. As a result, varying the “*n*” parameter has little to no impact on the outcome. In contrast, for larger chromophores or multichromophoric systems, this approach can be more effective, as it enables selective weighting of specific fragments or atomic types.

### 5.2 *H*-1,2,3-Triazole

The temporal evolution of the *S*_0_, *S*_1_, and *S*_2_ state populations, comparing different velocit*y*-direction adjustment methods, is shown in Figure 6a. Specifically, the full kinetic energy adjustment (**v**_full_), the NACV-direction adjustment (**d**), and three variants of excitation-weighted velocity rescaling (**v**_*w*_(*qeh/*1*TDM*)) for α = 1.00 are shown. Results for α = 0.50 can be found in Figure S3.

**Figure 6:**
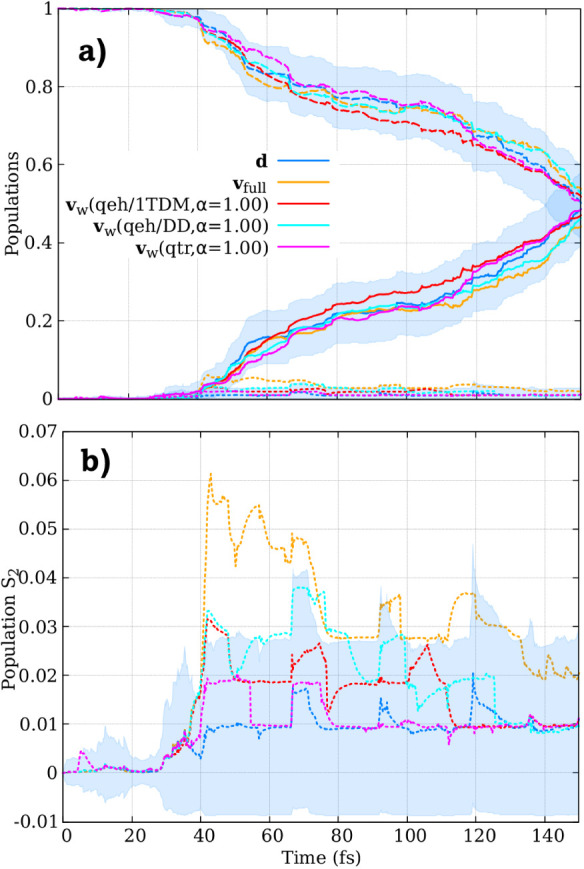
(a) Time-resolved state populations of 1*H*-1,2,3-triazole over the first 150 fs, computed using different velocity adjustment algorithms: velocit*y*-direction adjustment with full kinetic energy available (**v**_full_), NACV-direction adjustment (**d**), and three variations of excitation-weighted velocity rescaling (**v**_*w*_). The *S*_0_ state is represented by solid lines (−), *S*_1_ by dashed lines (- -), and *S*_2_ by dotted lines (···). Shaded regions indicate 95% confidence intervals (Γ) for the NACV-direction adjustment results, calculated as $\Gamma =p\pm 1.96\times \sqrt{\frac{p\left(1-p\right)}{N_{\mathrm{traj}}}}$ , where *p* is the state population and *N*_traj_ = 110 is the number of trajectories. (b) Magnified view of the *S*_2_ population across all approaches.

The *S*_1_ and *S*_0_ population curves exhibit an inverse trend: an initial plateau is observed during the first 30 fs of the simulation, which can be attributed to skeleton relaxation along the N2–N3 bond length alternation. Subsequently, the *S*_1_ population decays to the ground state, while a small fraction is transferred to the *S*_2_ state in all approaches. The magnified view of the *S*_2_ population (Figure 6b) reveals that the **v**_full_ method results in a slightly higher *S*_2_ population. This suggests that providing more kinetic energy facilitates back-hopping to the *S*_2_ state, compared to the other approaches.

The *S*_0_ and *S*_1_ populations over the entire time interval of the dynamics show that all approaches fall within the 95% confidence interval (shaded region) of the **d** dynamics, indicating consistency between these methods within statistical uncertainty. However, the *S*_2_ population for the **v**_full_ approach, for a significant portion of the dynamics, does not fall within the 95% confidence interval compared to the **d** simulations.

Analogous to fulvene, Table 3 presents the total number of hopping events (*S*_1_ → *S*_0_, *S*_2_ → *S*_0_, and *S*_2_ → *S*_1_ transitions), back-hopping events (*S*_0_ → *S*_1_, *S*_0_ → *S*_2_, and *S*_1_ → *S*_2_ transitions), and frustrated hopping events across all SH simulations in *H*-1,2,3-triazole. Additionally, we report the total number of trajectories analyzed, excluding those where total energy conservation was violated (total energy variation was superior to |0.50| eV) or where electronic structure calculations encountered issues. For a normalized comparison, values in parentheses represent the number of hopping, back-hopping, and frustrated hopping events per trajectory.

**Table 3:** Total Number of Hops (*N*_hop_), Back-Hops (*N*_back hop_), and Frustrated Hops (*N*_frust_) Events, along with the Total Number of Trajectories Considered (*N*_traj_), for Triazole under Different Velocity Adjustment Schemes^a^

	$N_{\mathrm{hop}}^{S_{1}\to S_{0}}$	$N_{\mathrm{back}\mathrm{hop}}^{S_{0}\to S_{1}}$	$N_{\mathrm{hop}}^{S_{2}\to S_{0}}$	$N_{\mathrm{back}\mathrm{hop}}^{S_{0}\to S_{2}}$	$N_{\mathrm{hop}}^{S_{2}\to S_{1}}$	$N_{\mathrm{back}\mathrm{hop}}^{S_{1}\to S_{2}}$	*N*_frust_	*N*_traj_
**d**	75 (0.682)	21 (0.191)	0 (0.000)	0 (0.000)	3 (0.027)	4 (0.036)	29 (0.264)	110
**v**_full_	81 (0.750)	33 (0.305)	1 (0.009)	1 (0.009)	15 (0.139)	17 (0.157)	1 (0.009)	108
**v**_*w*_(*qeh/*1*TDM*, α = 1.00)	73 (0.676)	22 (0.204)	0 (0.000)	0 (0.000)	11 (0.102)	12 (0.111)	15 (0.139)	108
**v**_*w*_(*qeh/*1*TDM*, α = 0.50)	72 (0.679)	21 (0.198)	1 (0.009)	0 (0.000)	8 (0.075)	9 (0.085)	12 (0.113)	106
**v**_*w*_(*qtr*, α = 1.00)	80 (0.748)	28 (0.262)	0 (0.000)	0 (0.000)	6 (0.056)	7 (0.065)	8 (0.075)	107
**v**_*w*_(*qtr*, α = 1.00)	72 (0.692)	25 (0.240)	0 (0.000)	0 (0.000)	8 (0.077)	8 (0.077)	11 (0.106)	104
**v**_*w*_(*qeh/DD*, α = 1.00)	72 (0.686)	24 (0.229)	1 (0.009)	0 (0.000)	9(0.086)	10 (0.095)	11 (0.105)	105
**v**_*w*_(*qeh/DD*, α = 0.50)	72 (0.692)	23 (0.221)	0 (0.000)	0 (0.000)	8 (0.077)	9 (0.086)	11 (0.106)	104

a Values in parentheses indicate the number of events per trajectory (e.g., $\frac{N_{\mathrm{hop}}^{S_{1}\to S_{0}}}{N_{\mathrm{traj}}}$ ).

The results indicate that the increased population of the *S*_2_ state observed with **v**_full_ primarily arises from a higher number of back-hopping events from *S*_1_ → *S*_2_ compared to the other approaches. Notably, **v**_full_ is the only approach where an *S*_0_ → *S*_2_ back-hopping event was observed. All **v**_*w*_ simulations showed improvements in the number of hopping and back-hopping events, yielding results closer to those obtained with the NACV-based approach (**d**) compared to **v**_full_. In particular, the **v**_*w*_(*qeh/*1*TDM*) method produced results that most closely aligned with those of **d**.

To evaluate the kinetic energy available for hopping, we extracted *S*_0_ → *S*_1_ back-hopping geometries and velocities from simulations using the **d** adjustment. As shown in Figure 7, the **v**_*w*_ approach generally overestimates the available kinetic energy compared to NACV-based calculations, though occasional underestimations occur. Nevertheless, **v**_*w*_ demonstrates better overall agreement than the other methods. While we did not perform dynamics using the **v**_*t*_(*qeh,* 1*TDM*) approach, we estimated its available kinetic energy for comparison. For both **d** and **v**_*w*_, the available kinetic energy remains below 1.0 eV, except for two geometries in the **d** method. In contrast, the full kinetic energy reservoir (**v**_full_) consistently yields values above 2 eV.

**Figure 7:**
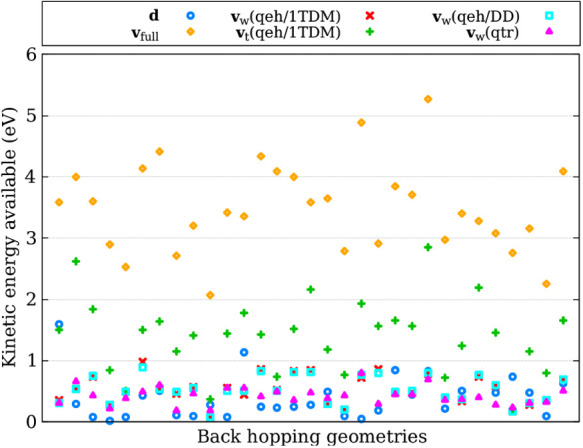
Kinetic energy available for hopping across different approaches for triazole. Each *x*-component corresponds to an *S*_0_ → *S*_1_ back-hopping geometry extracted from simulations using the NACV-direction adjustment. The kinetic energy is computed using four methods: **d**, **v**_*w*_, **v**_*t*_, and **v**_full_.

Finally, Figure 8 compares the average normalized atomic contribution of the NACV per atom for various *S*_0_ → *S*_1_ back-hopping geometries extracted from **d** adjustment simulations. The *x*-axis represents individual atoms. The *y*-axis shows the average coefficient values from eq 5 and the normalized NACV contribution for the 31 analyzed geometries. As seen in Figure 8, the **v**_*w*_(*qeh/DD*) approach most closely reproduces the atomic contributions obtained from **d**. Notably, this was also observed in fulvene.

**Figure 8:**
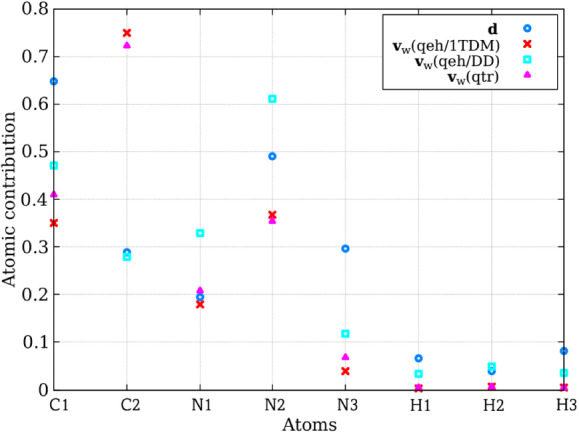
Comparison of the atomic contributions in velocity adjustment for NACV (**d**) and **v**_*w*_ in triazole dynamics. We present the average normalized contribution of the NACV per atom for different back-hopping geometries extracted from simulations using the **d** adjustment, compared with the average coefficients calculated according to eq 5 for the different **v**_*w*_ approaches. The *x*-axis represents individual atoms, where atoms (see Figure 1). The *y*-axis shows the average coefficient values and the normalized NACV contribution for the 31 *S*_0_ → *S*_1_ back-hopping geometries obtained through **d** calculations.

In summary, the investigated velocity adjustment methods show similar population dynamics over time for triazole, with minimal impact on the nuclear dynamics as well (see Section S3). However, the **v**_*w*_ method displayed a number of hops, back-hops, and frustrated hops that align more closely with the **d** compared to **v**_full_. Additionally, the kinetic energy available for hopping in **v**_*w*_ also shows much better agreement with **d** compared to **v**_full_.

## 6 Conclusions

In this study, we addressed the size-consistency issue in the surface hopping method by proposing two novel velocity rescaling approaches. Both methods adjust nuclear velocities based on atomic contributions to electronic transitions, which are derived from either the one-electron transition density matrix or the density difference matrix. The first method, *excitation-weighted velocity rescaling*, redistributes kinetic energy across atoms based on their individual contributions, using a normalized coefficient derived from the population analysis of the one-electron transition density matrix or density difference matrix. The second method, *excitation-thresholded velocity rescaling*, adjusts velocities only for atoms that exceed a predefined threshold.

The methods were tested to simulate the excited-state dynamics of fulvene and 1*H*-1,2,3-triazole. The results were compared against those resulting from the standard velocit*y*-direction adjustment, where kinetic energy is redistributed equally across all atoms and all atoms contribute to the kinetic energy available for hopping, as well as from the nonadiabatic coupling vector-direction adjustment. Our results show that *excitation-weighted velocity rescaling* produces results comparable to nonadiabatic coupling vector-direction adjustment. This approach reduces back-hopping events and provides a more balanced description of nonadiabatic transitions. The population dynamics for both fulvene and 1*H*-1,2,3-triazole show that all variants of the *excitation-weighted velocity rescaling* method fall within the 95% confidence interval of the nonadiabatic coupling vector-direction adjustment dynamics, indicating consistency between these methods within statistical uncertainty. Additionally, it improves the description of nuclear dynamics by allocating more kinetic energy to atoms actively involved in nonadiabatic transitions.

The *excitation-thresholded velocity rescaling* method was tested on fulvene and yielded results similar to the velocit*y*-direction adjustment. This is primarily because the system retains excess kinetic energy, leading to an excessive number of back-hopping events. Although this approach may not be superior for small organic molecules like fulvene, it may be worth exploring for larger chromophores or multichromophoric systems.

Overall, our study demonstrates that considering atomic contributions to electronic transitions provides a viable alternative for velocity adjustment in surface hopping, especially when nonadiabatic coupling vectors are unavailable or too computationally expensive to calculate.

## Supplementary Material



## Appendix

### Velocity adjustment along the nonadiabatic coupling direction

The total classical energy must be conserved during hopping between states. If the system hops from an old state to a new state, we must have

12
$$E_{\mathrm{kin}}^{\mathrm{old}}+E_{\mathrm{pot}}^{\mathrm{old}}=E_{\mathrm{kin}}^{\mathrm{new}}+E_{\mathrm{pot}}^{\mathrm{new}}$$

where *E*_kin_ represents the kinetic energy and *E*_pot_ represents the potential energy. The potential energy gap between the two states is defined as

13
$$\Delta E_{\mathrm{pot}}=E_{\mathrm{pot}}^{\mathrm{new}}-E_{\mathrm{pot}}^{\mathrm{old}}$$

Therefore, from the energy conservation equation, the kinetic energy variation is

14
$$\Delta E_{\mathrm{kin}}=E_{\mathrm{kin}}^{\mathrm{new}}-E_{\mathrm{kin}}^{\mathrm{old}}=-\Delta E_{\mathrm{pot}}$$

To adjust for the change in kinetic energy, the velocity of nucleus *A* is rescaled as

15
$$v_{A}^{\mathrm{new}}=v_{A}^{\mathrm{old}}+\gamma \frac{d_{A}^{\mathrm{old},\mathrm{new}}}{M_{A}}$$

where $d_{A}^{\mathrm{old},\mathrm{new}}$ is the velocity rescaling direction, in this case, in the direction of the nonadiabatic coupling vector between the states involved in the transition, and *M*_*A*_ is the nuclear mass. Substituting this into the kinetic energy equation (eq 14) leads to

16
$$a\gamma^{2}+b\gamma +\Delta E_{\mathrm{pot}}=0$$

where

17
$$a=\frac{1}{2}\underset{A}{\sum }\frac{1}{M_{A}}\left(d_{A}^{\mathrm{old},\mathrm{new}}\cdot d_{A}^{\mathrm{old},\mathrm{new}}\right)$$

18
$$b=\underset{A}{\sum }\frac{1}{M_{A}}\left(v_{A}^{\mathrm{old}}\cdot d_{A}^{\mathrm{old},\mathrm{new}}\right)$$

being the discriminant of this equation:

19
$$\Delta =b^{2}-4a\Delta E_{\mathrm{pot}}$$

Therefore, if Δ < 0, no real solution exists, and the hopping is frustrated. Otherwise, if Δ ≥ 0, the possible solutions are

20
$$\gamma =\left\{ \begin{array}{ll} \frac{-b+\sqrt{\Delta }}{2a}, & \mathrm{if}\left|-b+\sqrt{\Delta }\right|<\left|-b-\sqrt{\Delta }\right|\\ \frac{-b-\sqrt{\Delta }}{2a}, & \mathrm{otherwise} \end{array}\right.$$

Therefore, hopping is only allowed if:

21
$$\frac{1}{2\underset{A}{\sum }\frac{{\left(d_{A}^{\mathrm{old},\mathrm{new}}\right)}^{2}}{M_{A}}}{\left(\sum_{A}v_{A}^{\mathrm{old}}\cdot d_{A}^{\mathrm{old},\mathrm{new}}\right)}^{2}\geq \Delta E_{\mathrm{pot}}$$

From the conditions above, the kinetic energy available for hopping is

22
$$\Delta E_{\mathrm{kin}}\leq \frac{1}{2\underset{A}{\sum }\frac{{\left(d_{A}^{\mathrm{old},\mathrm{new}}\right)}^{2}}{M_{A}}}{\left(\sum_{A}v_{A}^{\mathrm{old}}\cdot d_{A}^{\mathrm{old},\mathrm{new}}\right)}^{2}$$

## Notes

Details on the active space used for the studied systems, additional results on the evolution of electronic populations over time, time evolution of some geometric parameters over time for 1*H*-1,2,3-triazole, and the simulated absorption spectra (PDF)
