# Supplementary material for: Velocity Rescaling in Surface Hopping Based on Atomic Contributions to Electronic Transitions
Source: J Chem Theory Comput. 2025 Aug 22;21(17):8278–90. doi: 10.1021/acs.jctc.5c00737 (PMC12424171; doi:10.1021/acs.jctc.5c00737)
Supplement: Supplementary file 1 [file ct5c00737_si_001.pdf]

Supplementary Information for:

"Velocity Rescaling in Surface Hopping Based on Atomic Contributions to Electronic Transitions"

Eduarda Sangiogo-Gil,*,† Lea M. Ibele,‡ Richard Bleyer,† and Leticia González*,†,¶

†*University of Vienna, Institute of Theoretical Chemistry, Währinger Str. 17, A-1090 Vienna, Austria*

‡*Aix Marseille University, CNRS, ICR, 13397 Marseille, France*

¶*Research Platform on Accelerating Photoreaction Discovery (ViRAPID), University of Vienna, 1090 Vienna, Austria*

E-mail: eduarda.sangiogo.gil@univie.ac.at; leticia.gonzalez@univie.ac.at

Contents

S1 Active space **2**

S2 Time-resolved state populations of 1*H*-1,2,3-triazole using v_w with $\alpha = 0.50$ **3**

S3 N2-N3 bond distance of 1*H*-1,2,3-triazole **4**

S4 Absorption spectra **5**

S5 Classical populations **6**

S1 Active space

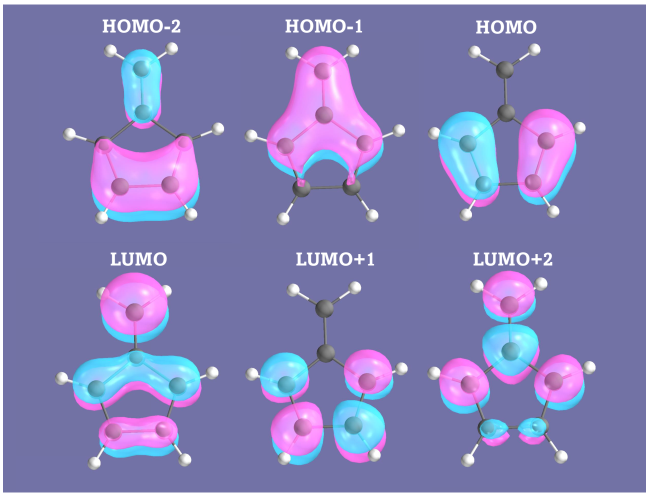

Figure S1: Fulvene's active space calculated at the SA(3)-CASSCF(6,6)/6-31G* level of theory.

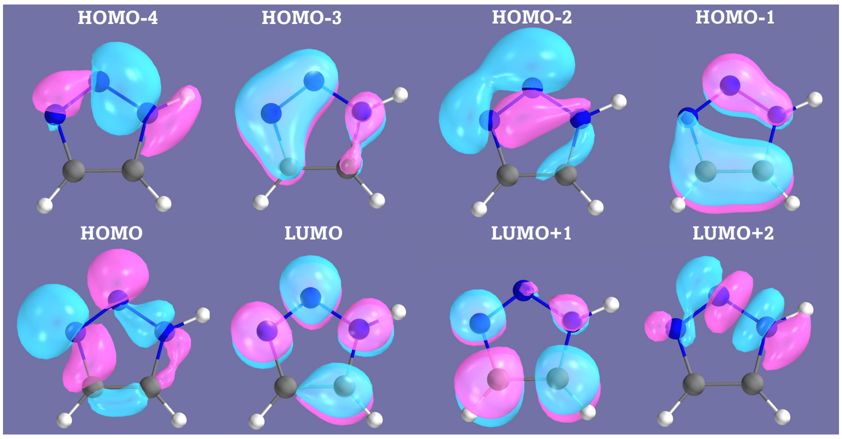

Figure S2: 1*H*-1,2,3-triazole's active space calculated at the SA(7)-CASSCF(10,8)/6-31G* level of theory.

S2 Time-resolved state populations of 1*H*-1,2,3-triazole using $\mathbf{v}_w$ with $\alpha = 0.50$

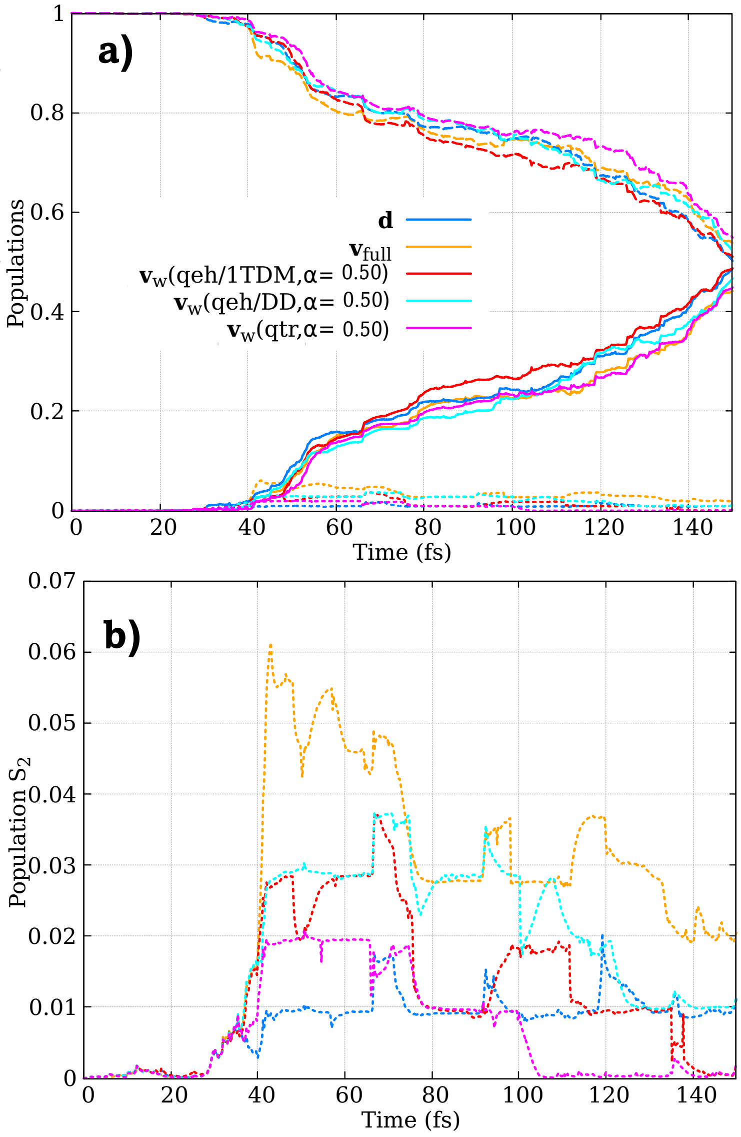

Figure S3: **a)** Time-resolved state populations of triazole over the first 150 fs, computed using different velocity adjustment algorithms: velocity-direction adjustment with full kinetic energy available ($\mathbf{v}_{\text{full}}$), NACV-direction adjustment ($\mathbf{d}$), and three variations of excitation-weighted velocity rescaling ($\mathbf{v}_w$). The S_0 state is shown as solid lines (–), S_1 as dashed lines (- -), and S_3 as dotted lines ($\cdots$). **b)** A magnified view of the S_2 state population across all approaches.

S3 N2-N3 bond distance of 1*H*-1,2,3-triazole

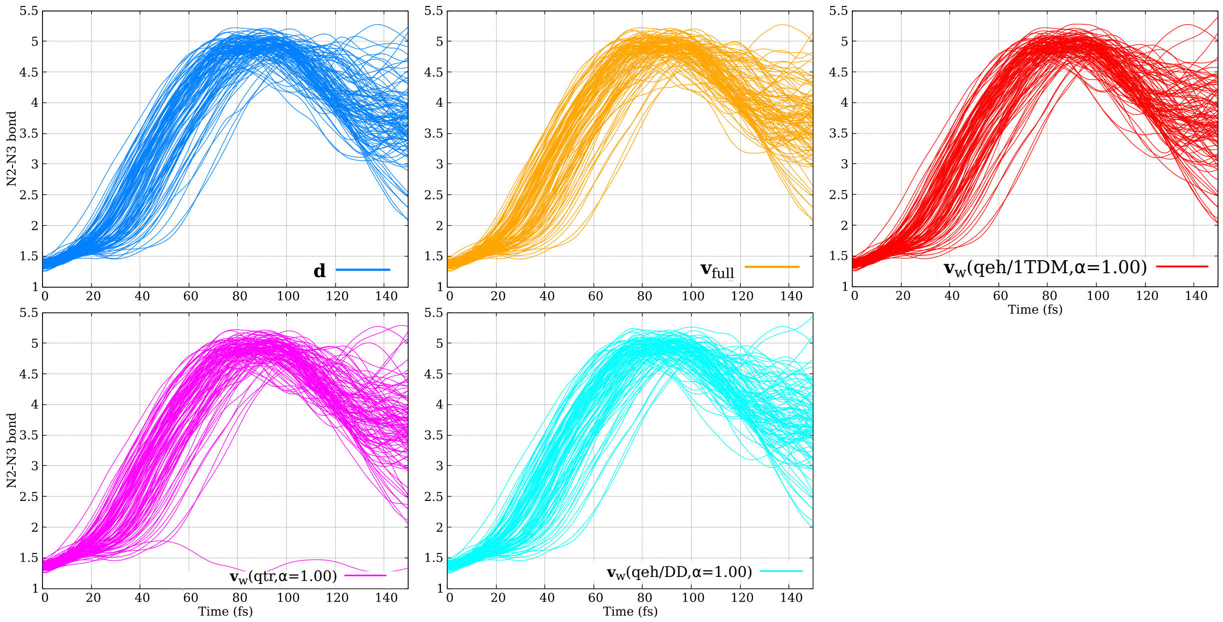

Figure S4: N2-N3 bond distance of triazole (see Fig. 6) for all individual trajectories using different velocity adjustment algorithms.

S4 Absorption spectra

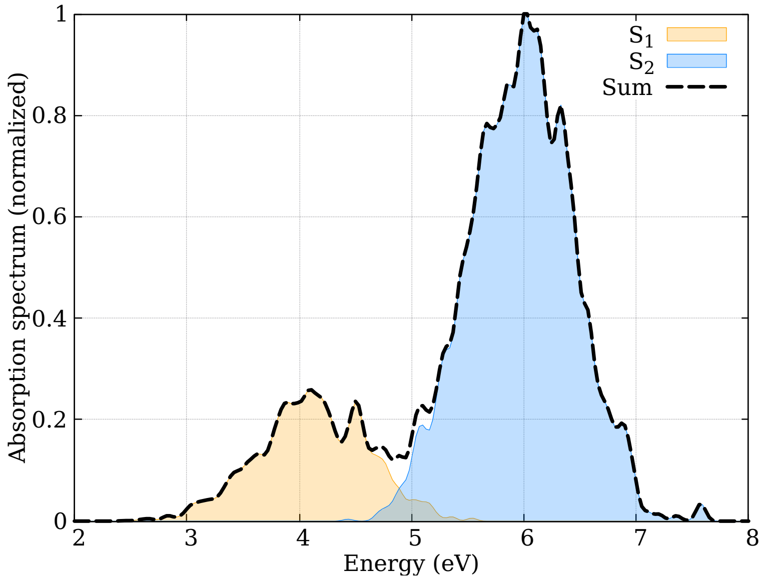

Figure S5: Absorption spectrum of fulvene computed using a Wigner distribution of nuclear positions for 2000 geometries at the SA(3)-CASSCF(6,6)/6-31G* level of theory.

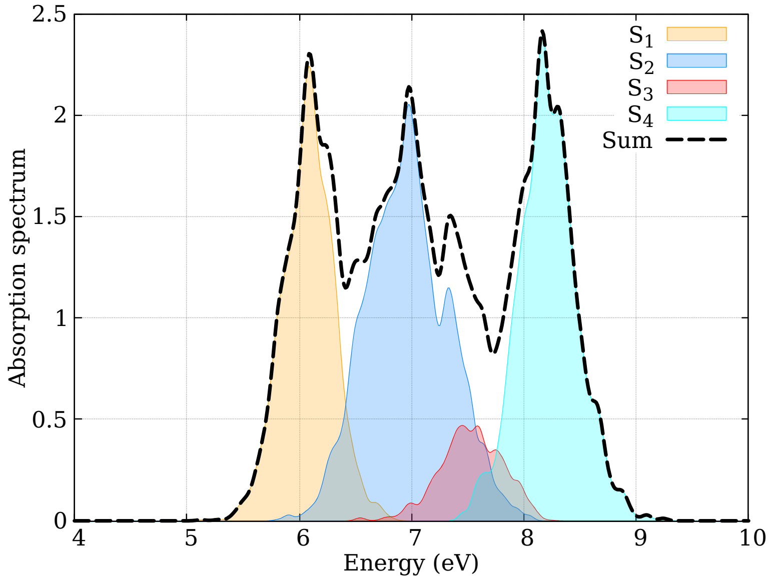

Figure S6: Absorption spectrum of 1*H*-1,2,3-triazole computed using a Wigner distribution of nuclear positions for 2000 geometries at the SA(5)-CASSCF(10,8)/6-31G* level of theory. The S_1 maximum agrees well with the experimental absorption spectrum is gas phase, which presents an S_1 maximum about 6.05 eV (*RSC Adv.*, **2019**, 9, 27361-27368, DOI: 10.1039/C9RA04235K).

S5 Classical populations

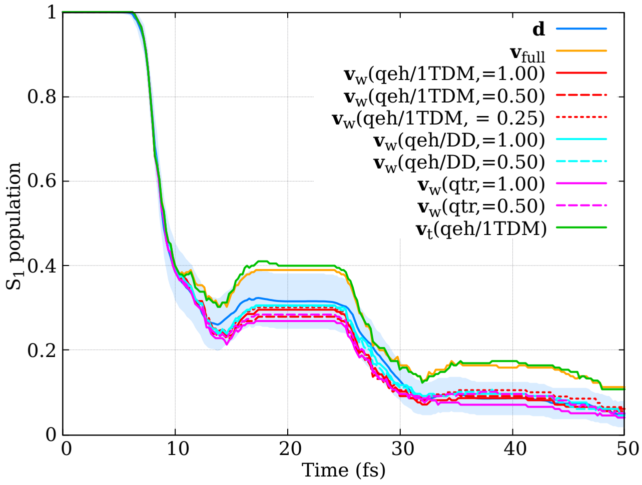

Figure S7: Time-resolved S_1 classical state population of fulvene over the first 50 fs, computed using surface hopping with different velocity adjustment algorithms: velocity-direction adjustment using the full kinetic energy available ($\mathbf{v}_{\text{full}}$), NACV-direction adjustment ($\mathbf{d}$), three different flavors of the excitation-weighted velocity rescaling ($\mathbf{v}_w$), and excitation-thresholded velocity rescaling ($\mathbf{v}_{c_{\text{max}}}$). Shaded regions indicate 95% confidence intervals (Γ) for the NACV-direction adjustment results, calculated as $\Gamma = p \pm 1.96 \times \sqrt{\frac{p(1-p)}{N_{\text{traj}}}}$, where p is the state population and $N_{\text{traj}} = 197$ is the number of trajectories.

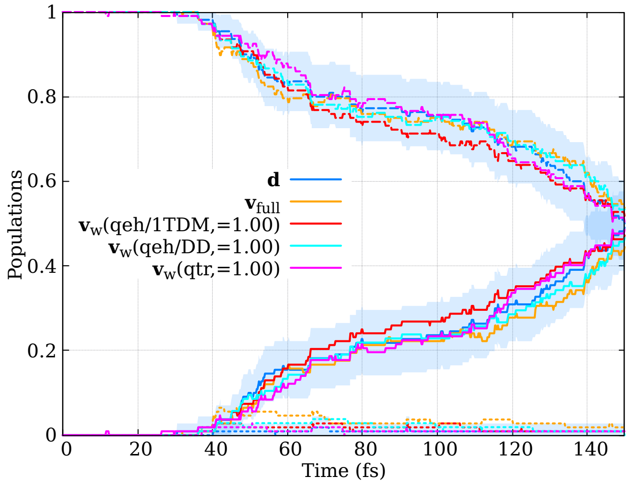

Figure S8: Time-resolved classical state populations of 1H-1,2,3-triazole over the first 150 fs, computed using different velocity adjustment algorithms: velocity-direction adjustment with full kinetic energy available ($\mathbf{v}_{\text{full}}$), NACV-direction adjustment ($\mathbf{d}$), and three variations of excitation-weighted velocity rescaling ($\mathbf{v}_w$). The S_0 state is represented by solid lines (–), S_1 by dashed lines (- -), and S_2 by dotted lines ($\cdots$). Shaded regions indicate 95% confidence intervals (Γ) for the NACV-direction adjustment results, calculated as $\Gamma = p \pm 1.96 \times \sqrt{\frac{p(1-p)}{N_{\text{traj}}}}$, where p is the state population and $N_{\text{traj}} = 110$ is the number of trajectories.
